# Parturition dysfunction in obesity: time to target the pathobiology

**DOI:** 10.1186/s12958-015-0129-6

**Published:** 2015-12-18

**Authors:** Nicole S. Carlson, Teri L. Hernandez, K. Joseph Hurt

**Affiliations:** Emory University, Nell Hodgson Woodruff School of Nursing, 1520 Clifton Road NE, Atlanta, GA 30322 USA; Department of Medicine, Division of Endocrinology, Metabolism, & Diabetes, College of Nursing, University of Colorado School of Medicine, 12801 E. 17th Ave, MS 8106, Aurora, CO 80045 USA; Department of Obstetrics & Gynecology, Divisions of Maternal-Fetal Medicine & Reproductive Sciences, University of Colorado School of Medicine, 12700 East 19th Ave, MS 8613, Aurora, CO 80045 USA

**Keywords:** Cesarean section, Cholesterol, Dystocia, Labor, Leptin, Meta-inflammation, Myometrium, Pregnancy, Uterus

## Abstract

Over a third of women of childbearing age in the United States are obese, and during pregnancy they are at increased risk for delayed labor onset and slow labor progress that often results in unplanned cesarean delivery. The biology behind this dysfunctional parturition is not well understood. Studies of obesity-induced changes in parturition physiology may facilitate approaches to optimize labor in obese women. In this review, we summarize known and proposed biologic effects of obesity on labor preparation, contraction/synchronization, and endurance, drawing on both clinical observation and experimental data. We present evidence from human and animal studies of interactions between obesity and parturition signaling in all elements of the birth process, including: delayed cervical ripening, prostaglandin insensitivity, amniotic membrane strengthening, decreased myometrial oxytocin receptor expression, decreased myocyte action potential initiation and contractility, decreased myocyte gap junction formation, and impaired myocyte neutralization of reactive oxygen species. We found convincing clinical data on the effect of obesity on labor initiation and successful delivery, but few studies on the underlying pathobiology. We suggest research opportunities and therapeutic interventions based on plausible biologic mechanisms.

**Electronic supplementary material:**

The online version of this article (doi:10.1186/s12958-015-0129-6) contains supplementary material, which is available to authorized users.

## Background: The clinical phenotype of labor in obese women

Over 30% of childbearing age women in the United States are obese (body mass index [BMI] ≥ 30 kg/m^2^), with higher rates among racial and ethnic minority groups (31.8 % overall, 35.8 % among Hispanic and 55.8 % among non-Hispanic Black women) [1]. Obesity is associated with a number of pregnancy complications including increased risk of gestational diabetes (OR 2.83), gestational hypertension/pre-eclampsia (OR 2.68) [2], and maternal depression (OR 1.43) [3]. Maternal obesity also increases fetal risks for congenital anomalies [4] and macrosomia (birth weight > 4,500 g) [5], and for lifetime risks of heart disease [6], diabetes, and obesity [4] as an adult.

The onset of parturition in obese women is frequently delayed. Without induction, obese women are nearly twice as likely as normal-weight women to have prolonged pregnancy (≥41 weeks gestation), particularly with BMI of 35 kg/m^2^ or higher [7–9]. In contrast, underweight women (BMI < 17 kg/m^2^) are more than twice as likely to deliver preterm in spontaneous labor [10]. Prolonged pregnancy is concerning because there is a two-fold increased risk of third-trimester stillbirth in obese women [11]. Interestingly, obese women are also more likely than normal-weight women to deliver preterm [12, 13], although 60% of those early births are medically indicated [13] so the majority of these early deliveries are likely related to obesity-associated pathology.

During labor, the progress of cervical dilation in obese women is slower than in normal-weight women [14–17], a complication known as labor dystocia [18]. In two large prospective cohorts, increasing maternal BMI had a clinically relevant dose relationship with protracted labor (Table 1) [19, 20]. The time to full cervical dilation in morbidly obese (BMI ≥ 40 kg/m^2^) mothers was significantly longer than normal weight women, regardless of parity. Even in healthy obese women (without diabetes, chronic hypertension, or cardiovascular disease) the increased risk for slow cervical dilation (OR 3.9) and cesarean section (OR 3.2) persists [21]. We found no studies comparing labor outcomes directly in obese women with or without obesity-related metabolic dysfunction.

**Table 1:** Obese women demonstrate abnormally slow cervical dilation in first stage labor

Number previous vaginal births	Study	BMI <25.0	BMI 25.0-29.9	BMI 30.0-34.9	BMI 35.0-39.9	BMI ≥40	*p* value
Zero	Kominiarek et al., 2011 [19]						
	Median	5.4 hrs	5.7 hrs	6.0 hrs	6.7 hrs	7.7 hrs	<0.0001
	(95 % ile)	(18.2 hrs)	(18.8 hrs)	(19.9 hrs)	(22.2 hrs)	(25.6 hrs)	
	Norman et al., 2012 [20]						
	Median	4.6 hrs	5.0 hrs	5.5 hrs		6.7 hrs	<0.01
	(95 % ile)	(14.4 hrs)	(15.7 hrs)	(17.3 hrs)		(21.2 hrs)	
One	Kominiarek et al., 2011 [19]						
	Median	4.6 hrs	4.5 hrs	4.7 hrs	5.0 hrs	5.4 hrs	<0.0001
	(95 % ile)	(17.5 hrs)	(17.4 hrs)	(17.9 hrs)	(19.0 hrs)	(20.6 hrs)	
	Norman et al., 2012 [20]						
	Median	3.3 hrs	3.9 hrs	4.3 hrs		5.0 hrs	<0.01
	(95 % ile)	(12.6 hrs)	(15.1 hrs)	(16.5 hrs)		(19.2 hrs)	

Adjusted duration of labor from 4–10 centimeters cervical dilation by BMI at the time of delivery. Data are median and 95%ile hours in labor

Kominiarek’s median duration adjusted for age, height, race, gestational age, diabetes, induction, augmentation, epidural (first stage), operative vaginal delivery, and birthweight (*N* = 118,978)

Norman’s median duration adjusted for induction, race, birth weight > 4,000 g (*N* = 5,204)

Obese women are more likely to be admitted to the hospital at earlier cervical dilation, and to undergo additional intervention to facilitate labor [22, 23], including administration of synthetic oxytocin (Pitocin; Table 2). Currently, a standard Pitocin regimen is typically used regardless of BMI [15] though evidence suggests that higher median dose and longer duration may be necessary for labor progress in obese women [24]. Despite increased clinical intervention, obese women are two to three times more likely than normal-weight women to have unplanned cesarean section (Table 2) [17, 25], a risk that persists after controlling for obesity-associated co-morbidities [16, 17]. Post-operative complications are also more common among obese women, including infection (18.8 % BMI > 45 kg/m^2^ vs. 7.5 % normal-weight) [26, 27], postpartum hemorrhage (32.6 % morbidly obese vs. 4.9 % normal-weight), and prolonged hospitalization (34.9 % morbidly obese vs. 2.3 % normal-weight) [28].

**Table 2:** Increased intrapartum interventions and increased risk for cesarean delivery for obese parturients

Intrapartum intervention	Study	BMI category	Odds of use in labor, OR (95 % CI)
Induction of Labor	Scott-Pillai et al., 2013 [142]	Overweight	1.2 (1.1-1.3)^g, h^
		Obese	1.3 (1.2-1.5)^g, h^
		Obese II	1.4 (1.3-1.6)^g, h^
		Morbid obese	1.6 (1.3-1.9)^g, h^
	Garabedian et al., 2011 [143]	Overweight	1.51 (1.42-1.60)^a^
		Obese	2.00 (1.87-2.15)
		Obese II	2.36 (2.16-2.58)
		Obese III	3.66 (3.30-4.01)
		BMI 40–49.9	3.51 (3.15-3.91)
		BMI ≥ 50	5.25 (3.87-7.10)
	Bhattacharya et al., 2007 [9]	Overweight	1.3 (1.2-1.4)^f^
		Obese	1.8 (1.6-2.0)^f^
		Morbid obese	1.8 (1.3-2.5)^f^
Artificial Rupture of Membranes prior to 6 cm cervical dilation	Jensen, Agger, Rasmussen, 1999 [144]	Overweight	1.63 (1.18-2.25)^g,b^
		Obese	1.97 (1.20-3.25)^g, b^
Oxytocin Augmentation of Labor	Garabedian et al., 2011 [143]	Overweight	1.38 (1.28-1.49)^a^
		Obese	1.87 (1.70-2.06)
		Obese II	2.05 (1.79-2.34)
		Obese III	3.02 (2.57-3.55)
		BMI 40–49.9	3.00 (2.53-3.56)
		BMI ≥ 50	3.21 (1.97-5.23)
	Abenhaim & Benjamin, 2011 [145]	Overweight	1.31 (1.15-1.49)^g^
		Obese	1.51 (1.31-1.75)^g^
		Morbid obese	3.05 (1.89-4.94)^g^
	Vahratian, 2005 [146]	Overweight	Significantly higher use in both categories^c^
		Obese	
	Jensen, Agger, Rasmussen, 1999 [144]	Overweight	1.59 (1.22-2.06)^f,^ ^b^
		Obese	1.98 (1.28-3.05)^g,^ ^b^
Unplanned Cesarean Delivery	Vinturache et al., 2014 [147]	Overweight	
		Spontaneous labor	1.1 (0.6-1.8)
		Induced labor	1.2 (0.7-2.0)
		Obese	
		Spontaneous labor	1.5 (0.7-3.0)
		Induced labor	2.2 (1.2-4.1)^f^
	Scott-Pillai et al., 2013 [142]	Overweight	1.4 (1.2-1.5)^g, h^
		Obese	1.6 (1.4-1.8)^g, h^
		Obese II	1.8 (1.5-2.2)^g, h^
		Morbid obese	1.9 (1.4-2.5)^g, h^
	Green & Shaker, 2011 [148]	BMI >35	No sig difference once adjusted for IOL^c^
	Garabedian et al., 2011 [143]	Overweight	1.44 (1.38-1.50)^g^
		Obese	1.96 (1.86-2.06)^g^
		Obese II	2.32 (2.17-2.47)^g^
		Obese III	3.66 (3.39-3.95)^g^
		BMI 40–49.9	3.53 (3.26-3.82)^g^
		BMI ≥ 50	4.99 (4.00-6.22)^g^
	Abenhaim & Benjamin, 2011 [145]	Overweight	1.07 (0.80-1.43)^d^
		Obese	
		Morbid obese	
	Cedergren, 2009 [149]	Overweight	1.09 (0.91-1.31)
	*Due to obstructed labor*	Obese I	1.56 (1.14-2.14)^f^
		Obese II	1.33 (0.72-2.46)
		Morbid obese	1.79 (0.65-4.92)
	Cedergren, 2009 [149]	Overweight	1.50 (1.42-1.59)^g^
	*Due to ineffective uterine contractility*	Obese I	2.14 (1.96-2.34)^g^
		Obese II	2.72 (2.35-3.16)^g^
		Morbid obese	3.98 (3.14-5.04)^g^
	Bhattacharya et al., 2007 [9]	Overweigh	1.5 (1.3-1.6)^f^
		Obese	2.0 (1.8-2.3)^f^
		Morbid obese	2.8 (2.0-3.9)^f^
	Sukalich, Mingione, Glantz, 2006 [150]	Obese	1.07 (1.05-1.09)^f^
	Vahratian, 2005 [146]	Overweight	1.2 (0.8-1.8)^f,^ ^e^
		Obese	1.5 (1.05-2.0)^f^
	Jensen, Agger, Rasmussen, 1999 [144]	Overweight	1.69 (1.06-2.68)^f,^ ^b^
		Obese	1.91 (0.94-3.86)^b^

Updated from Carlson & Lowe [111]. Odds ratios are for comparison with women of normal BMI

^a^Significance not computed

^b^OR and CIs calculated from frequency tables provided in manuscript

^c^OR and CI not provided

^d^Not significant when adjusted for known confounders (maternal age, parity, previous c/s, DM, GDM, hypertension, preeclampsia, cervix on admit, IOL, birthweight, gestational age) and for labor management differences (use of epidural analgesia, oxytocin, forceps, vacuum)

^e^Adjusted risk ratio reported

^f^Significant at *p* < .05

^g^Significant at *p* < .001

^h^Adjusted for age, parity, social deprivation, smoking, and year of birth

Reduced gestational weight gain in obese women improves maternal and fetal outcomes in pregnancy, and the Institute of Medicine has recommended revised prenatal care guidelines [29, 30]. Lower weight gain decreases the risk of preeclampsia, cesarean delivery, large for gestational age neonates, and small for gestational age neonates [29]. There are no data on the safety and outcomes of intentional weight loss for obese pregnant women, and that approach is controversial due to concerns for fetal growth and maternal ketosis [31]. In studies using obese mice, maternal weight loss alters epigenetic signatures in the offspring [32], emphasizing the need to understand possible influences of altered prenatal goals.

We need new approaches to improve birth outcomes in obese women, who have a unique parturition phenotype [24, 33, 34]. Biologic mechanisms linking obesity to dysfunctional labor, independent of obesity-associated comorbidities, are largely unknown. In this review, we first outline normal parturition physiology and relevant obesity pathophysiology. We then summarize studies from human and animal models identifying the possible molecular basis for differences in labor preparation, uterine contraction/synchronization, and labor endurance in obese women. We suggest opportunities for future investigation, and possible therapeutic targets and approaches to improve birth outcomes.

## Overview of normal parturition physiology relevant to obesity

Human parturition can be described by myometrial phases: quiescence, preparation (activation), labor (contractions), and recovery (involution) [35]. Ninety-five percent of human gestation is spent in quiescence, when the myometrium is not contracting and the cervix is closed. In the preparation phase, the myometrium expresses contractile-associated proteins (CAPs) including oxytocin receptor (OTR), prostaglandin F receptor, and connexin-43, and the cervix softens and shortens [36]. In active labor, synchronized uterine contractions dilate the cervix for delivery of the fetus. In recovery, the uterus and cervix involute and remodel for future pregnancy. Specific signals regulate each phase of parturition.

### Preparation for parturition

Preparation for parturition is initiated during the final 12 weeks of pregnancy when placental corticotropin-releasing hormone (pCRH) increases dramatically, leading some investigators to characterize pCRH as a gestational clock [37]. Simultaneously, maternal plasma CRH binding protein decreases, allowing increased free pCRH to activate myometrial CRH-receptors that switch from an isoform that enforces quiescence to an isoform that activates CAP expression [38]. pCRH also stimulates fetal pituitary adrenocorticotropic hormone (ACTH) release, increasing fetal adrenal cortisol production and maternal myometrial expression of cyclooxgenase-2 (COX-2) [35]. COX-2 synthesizes prostaglandins (PG) E_2_ and PGF_2alpha_ to promote myometrial activation. Fetal cortisol increases placental pCRH production in a feed-forward loop that stimulates fetal production of dehydroepiandrosterone (DHEA) which is converted to estriol by placental enzymes [39]. Estriol, the dominant placental estrogen, increases myometrial sensitivity to oxytocin and PGs, and stimulates oxytocin release from choriodecidual tissues [40].

In humans, progesterone does not decline dramatically at term, but serum progesterone increases at a slower rate while estradiol or estriol production accelerates, leading to an elevated estrogen to progesterone ratio. These changes are sometimes characterized as functional progesterone withdrawal [35]. Further, and perhaps more important, progesterone receptor (PR) expression changes from a predominance of PR-B to more PR-A receptors [35], thereby activating a different set of genes [41]. PR-B mediates the primary pro-gestational (i.e., quiescence) function of progesterone while the truncated N-terminal of PR-A prevents recruitment of coactivators and alters myometrial progesterone gene activation. Thus, the PR isoform switch reduces progesterone’s pregnancy maintenance function and facilitates the molecular program leading to myometrial contractility. Other factors suggested to modulate the myometrial labor program include epigenetic regulation of pCRH gene expression [42], NFkappaB inflammatory blockade of PR responsive genes [43], recruitment of inflammatory macrophages to the myometrium [44], and perhaps increased cell-free fetal DNA in the maternal circulation [45].

The cervix shortens and ripens for several weeks before labor in response to PGE_2_ and PGF_2alpha_, released by fetal membranes [35]. Cervical extracellular collagen disperses, water content increases, and fibrous stroma degrades. Matrix metalloproteinases (MMPs), released by cervical stromal fibroblasts and smooth muscle cells, break down cervical stromal proteoglycans [46, 47]. In addition, cervical epithelial and stromal cells undergo apoptosis, decreasing the cellular content of the laboring cervix [48]. The amnion and chorion overlying the internal cervical os degrade before labor, with increased apoptosis leading to rupture of membranes [49]. Disruption of fetal membranes releases amniotic fluid that exposes decidua and myometrium to PGE2 and oxytocin, enhancing labor activation [35].

### Contraction & synchronization in parturition

Phasic uterine contractions are a hallmark of active labor, accompanied by progressive cervical dilation. Individual myocytes contract when actin and phosphorylated myosin interact via ATP-consuming cross-bridge cycling [50] triggered by increased intracellular calcium ion (Ca^2+^). Prior to parturition, Ca^2+^ efflux and intracellular sequestration lead to decreased intracellular Ca^2+^ and myocyte relaxation. Low resting Ca^2+^, along with potassium (K^+^) channel opening and plasma membrane repolarization promote the quiescent state. During labor, myosin phosphorylation is initiated by myocyte depolarization leading to calcium influx, primarily via L-type Ca^2+^ channels. Oxytocin signaling via the oxytocin receptor (OTR) promotes more frequent and forceful uterine contractions and release of PGs from the decidua [35, 51]. The posterior pituitary releases pulsatile oxytocin, and decidual and placental tissues produce oxytocin continuously [35, 52]. During late gestation, OTRs increase sharply in the myometrium and decidua as the primary mediator of oxytocin response in labor. The basic biology of myocyte contractility has been reviewed in detail elsewhere [35, 50, 53, 54].

During labor, myocyte contraction is coordinated, starting in the uterine “pacemaker” at the fundus and spreading toward the cervix [55, 56]. Neighboring myocytes become connected via plasma membrane gap junctions that assemble immediately before labor [57]. These low-resistance pores formed by connexins (e.g., connexin-43), allow action potentials to travel freely from cell to cell, transforming the uterus into a functional syncytium [58]. Stronger and longer originating action potentials result in organized uterine contractions of increasing force [59, 60].

### Uterine endurance in parturition

During labor, the uterus must maintain sufficient strength and duration of contraction, or endurance, to expel the fetus and maintain postpartum hemostasis. During contractions, ATP hydrolysis releases protons, producing transient acidification [50]. Forceful contractions occlude blood vessels in the uterine wall, causing repetitive ischemia and reperfusion that leads to periodic acidification due to anaerobic lactic acid production [50, 61]. Cyclic hypoxia/reperfusion also produces reactive oxygen species (ROS) [62]. Myocyte regulatory mechanisms counteract the lower pH (e.g., hypoxia-resistant lactate dehydrogenase isoform) and elevated ROS (e.g., Glutathione Peroxidase [GSHPx]), maintaining labor endurance [50, 63].

## Overview of obesity physiology relevant to pregnancy

Nearly 70 % of obese patients exhibit metabolic dysregulation with changes in circulating hormones [64–66] and free fatty acids (FFA). For this reason, obesity results in altered physiology that may influence normal parturition pathways.

### Circulating molecules in obesity

Increasing BMI alters the secretion of a range of hormones from adipose tissue. Highly relevant to pregnancy is leptin, an adipokine secreted primarily by white adipose tissue. Leptin suppresses appetite, stimulates adipocyte hypertrophy, and increases FFA oxidation [67, 68]. Free leptin rises with BMI and increased adiposity, but obese individuals also exhibit impaired satiety feedback. This “leptin resistance” may be the result of chronic inflammatory mediators that disrupt normal hypothalamic homeostasis [67, 69, 70]. The placenta also secretes leptin [67, 71], peaking in the second trimester. Leptin is significantly increased in obese compared to normal-weight pregnant women [72]. In contrast, soluble leptin receptor decreases linearly with increasing BMI in pregnancy, leading to high serum free leptin in obese pregnant women [67].

Four other adipokines may be of importance. Apelin, secreted by adipose tissue and placenta [73, 74], is elevated in obese pregnant women and associated with insulin resistance [75]. Physiologically, apelin causes hypotension, regulates fluid homeostasis intake, and is produced in response to insulin release [73]. Visfatin increases in pregnant women near labor [76]. Visfatin is highly expressed by visceral adipose cells, and activates insulin receptors. Ghrelin, in contrast, is inversely related to maternal BMI. It stimulates insulin secretion, and regulates food intake and fat utilization during pregnancy [77, 78]. The most abundant adipokine is adiponectin, which regulates energy metabolism by increasing glucose uptake, lipid catabolism, and insulin sensitivity [79]. Adiponectin is decreased among pregnant women with higher BMI and those with more pronounced dyslipidemia [80].

Plasma cholesterol increases with obesity and also in pregnancy. Plasma lipids and lipoproteins increase throughout pregnancy, supplying nutrients to the fetus and supporting fetal cholesterol biosynthesis [80]. In reproductive age females, hypercholesterolemia increases with BMI beyond that associated with normal pregnancy [81]. Perhaps more importantly, obese insulin resistant women (i.e., more metabolically dysfunctional) show a shift to an atherogenic lipoprotein phenotype, including increased plasma triglycerides and small low density lipoproteins (LDL) [80, 82], that is associated with gestational diabetes [83], ectopic fat deposition [84], and atherosclerosis [85].

Obesity also alters vitamins, minerals, and cofactors. For example, low vitamin D levels are found more often among obese and overweight individuals, and associated with chronic disorders like cardiovascular disease and diabetes [86]. Low folic acid levels are similarly linked to obesity, resulting in increased neural tube defects in offspring of obese women [87].

### FFA storage and meta-inflammation

In pregnancy, obese women have higher triglyceride and FFA levels than normal-weight pregnant women. When FFA levels overwhelm adipocyte storage capacity, excess FFA is sequestered as triglyceride in new adipocytes or within fat droplets in non-adipose tissues (ectopic fat) [65]. As FFA levels increase further, they may circulate and be used instead of glucose for cellular metabolism [88]. Mitochondrial lipid metabolism produces ROS that can damage cellular structures and impair insulin responses, a process called lipotoxicity. Normally, cells neutralize ROS with cellular antioxidants, but with increased FFA, that capacity is overwhelmed, and ROS cause RNA/DNA damage, protein carbonylation, and eventual apoptosis [63, 89, 90]. ROS-mediated cell damage releases inflammatory mediators (e.g., interleukin-6, interleukin-1ß, tumor necrosis factor-α), creating chronic low-grade inflammation throughout the body known as meta-inflammation [91, 92], which further increases ROS production [90].

Insulin is crucial for glucose uptake, glycogen synthesis, lipogenesis, and cell growth, and stimulates adipose uptake and storage of FFA [93]. Insulin resistance is normal in human pregnancy, a necessary adaptation to ensure appropriate maternal nutrient shunting to the fetus [94]. As insulin resistance exacerbates the FFA storage problem, pregnancy may worsen obesity-related ROS damage and meta-inflammation [93].

## Methods: a literature search for interactions between obesity and parturition signaling

Considering the known physiology described above, we explored the experimental evidence for interactions between the physiology of obesity and normal parturition signaling. Our primary question was: What biologic mechanisms could be responsible for parturition dysfunction in obesity? In June 2015, we performed a comprehensive literature review using PubMed, Google Scholar, Web of Science, and MEDLINE databases with the primary medical subject heading (MeSH) search terms: obesity, labor, parturition, and pregnancy. Additional search terms included: BMI, uterus, myometrium, dystocia, mechanism, and dysfunction. We considered all original research in the English language from any year. We also considered relevant articles from citations in these publications. After initial screening, we obtained full-text articles for evaluation of content, quality, and relevance and included all studies relevant to potential interactions between physiologic changes of obesity and dysfunctional labor (Additional file 1: Table S1).

## Review of evidence: biologic mechanisms of labor dysfunction in obesity

**Fig. 1:**
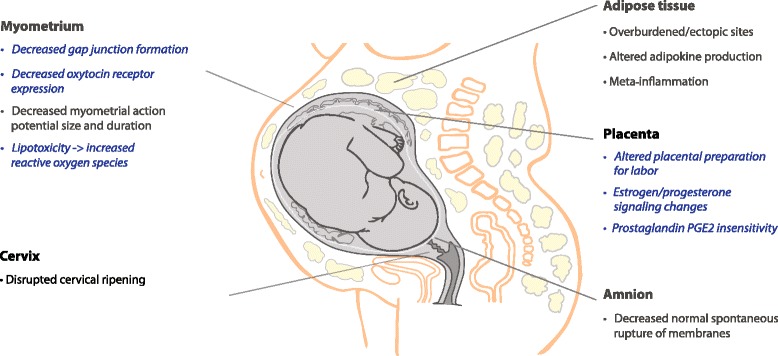
Overview of Obesity-Related Biologic Dysfunction of Labor. PGE_2_ = prostaglandin E2 (dinoprostone; naturally occurring prostaglandin). Blue italicized script = Proposed mechanism or mechanism with limited evidence

We identified 31 studies for review (Additional file 1: Table S1) and have grouped them into three categories: labor onset, contraction/synchronization, and endurance. They include human, animal, and cell culture studies. Figure 1 summarizes interactions between obesity and parturition. Some mechanisms were corroborated by multiple investigators, while others involve only early work and suggested hypotheses.

### Changes in labor preparation due to obesity

#### Placental function

Obesity increases placental weight and hypertrophy, but reduces the fetal/placental weight ratio (sometimes referred to as “placental efficiency”) [95]. A high fat diet decreases uterine blood flow, potentially leading to placental relative hypoxia and trophoblast dysfunction [96]. Placental amino acid transport is reduced in women with normal birthweight infants [97], but increased in obese mice [98] and in women with large babies [99]. Altered placental steroid hormone biosynthesis in obese pregnant women has not been established, but obesity is associated with lower mid- and late-pregnancy pCRH [100, 101]. Decreased pCRH together with changes in placental structure/function could alter estrogen/progesterone production, metabolism, or ratio resulting in delayed onset of parturition [102]. We found no studies on the changes in placental parturition steroid hormone signaling with obesity that might account for altered parturition timing.

#### Elevated leptin

Leptin stimulates PGE_2_ release from placental and adipose tissue via inflammatory signaling pathways [103]. Chronically elevated PGE_2_ in late pregnancy among obese women with meta-inflammation could decrease the sensitivity of maternal tissues to PGE_2_ during labor activation, a finding that has been documented clinically [104] and is supported by the known elevated PGE_2_ in obesity [105, 106]. Insensitivity to PGE_2_ during labor induction is linked to failed functional progesterone withdrawal [107]. Thus, the chronic inflammatory state of obese women could impede functional progesterone withdrawal and PGE_2_ activation. There is no current evidence examining changes in myometrial PRB/PRA switching with obesity.

Leptin also disrupts in vitro collagen degradation by MMPs, as well as cervical cell apoptosis [108, 109], two effects that may inhibit cervical ripening in obese women. Further, leptin stimulates cervical collagen synthesis in late pregnancy [108, 151], possibly explaining the decreased cervical ripening at term in obese women that we see clinically [110]. High circulating leptin in the second trimester may also inhibit fetal membrane weakening through decreased membrane apoptosis [109], thereby inhibiting spontaneous rupture of membranes in obese women [49]. The clinical finding that there is an increased requirement for artificial rupture of membranes with increasing BMI supports this hypothesis (Table 2) [111].

### Changes in labor contraction/synchronization due to obesity

#### Adipokines

Leptin exerts an inhibitory effect on spontaneous and oxytocin-stimulated myometrial contractions in vitro [112] however, the second trimester serum leptin level does not explain the risk of failed first-stage labor in an adjusted model [113]. To our knowledge, there are no published reports on labor outcome related to third-trimester leptin levels. Visfatin also decreases both spontaneous and oxytocin-induced contractions [76]. There are no data on whether adiponectin influences myometrial contractility [114]. Apelin also inhibits spontaneous and oxytocin-induced myometrial contractions in organ bath, but has not been evaluated clinically [73]. Ghrelin may stimulate myometrial contractions [114], although there are conflicting reports [78].

#### Cholesterol

Cholesterol supports plasma membrane channel function in cholesterol-rich “lipid rafts” [115]. Smooth muscle cells, including myocytes, are rich in a particular type of lipid raft, the caveolae [116], on which potassium channels such as Maxi-K and human ether-a-go-go–related gene (hERG) K^+^ channels cluster [60, 117]. These hyperpolarizing, and therefore pro-relaxant channels, are more active with the hypercholesterolemia that is more common among obese than normal-weight women. Both estrogen receptors [118] and oxytocin receptors [119] associate with myocyte membrane caveolae, but it is unknown whether maternal hypercholesterolemia alters the stability or function of these receptors. Cholesterol and LDL reduced contraction force and frequency in human and rodent myometrial strips, effects that were not alleviated by exogenous oxytocin [120, 121]. Mice with dysfunctional hepatic cholesterol clearance exhibited reduced oxytocin responses and abnormal labor without pup expulsion [122]. In humans, elevated maternal cholesterol at 14–16 weeks gestation is not a risk factor for first stage labor dystocia leading to cesarean section [123]. We found no studies that evaluated labor progress or outcomes by maternal cholesterol or maternal triglyceride levels near the time of birth.

#### Oxytocin receptors (OTR)

Obese women require more Pitocin for labor induction, even controlling for neonate size, parity, and epidural anesthesia [33, 124, 125]. Existing reports on obesity-dependent changes in OTR are contradictory [152]. In one study, OTR mRNA was decreased in myometrial biopsies from term pregnancies in women with higher BMI at delivery [126], but another group found no change in OTR gene or protein expression with increasing BMI [127], though greater than 30% of those subjects were laboring. Biopsy location, timing in pregnancy, and stage of labor could all influence OTR results [51], and women with protracted labor and delayed transition to active labor have different OTR gene polymorphisms than those with efficient labor [128]. Thus, labor progress is demonstrably sensitive to OTR expression, suggesting the important question of whether altered OTR is in part responsible for delayed labor in obese women. Genome-wide association studies have not evaluated interactions between obesity, OTR, and labor outcomes.

#### Gap junctions

We found no human studies investigating uterine connexin-43 expression in obesity or with labor dystocia. Uterine biopsies from a mixed-weight sample of women with prolonged labor have decreased connexin-43 mRNA compared to normal labor [58]. Rats fed a high-fat, high-cholesterol diet during pregnancy showed significantly lower connexin-43 expression compared to animals eating regular chow, although contractility was not examined [129].

### Changes in uterine endurance in labor due to obesity

Women with slow labor have lower oxygen saturation, increased myometrial lactate, and more acidic capillary pH compared to normal controls (pH 7.35 in dysfunctional labor vs. 7.48 in normal labor) [130]. One labor dystocia theory posits that lower pH and increased ROS cause unorganized and ineffective contractions, and an individual’s capacity to buffer pH and neutralize ROS predicts labor dystocia [130]. It is possible that obesity-related lipotoxicity when combined with normal myometrial ROS production during labor leads to excess ROS that might cause labor dystocia. Increased pre-pregnancy BMI is associated with excessive placental ROS and decreased ATP production [131]. However, high-fat/cholesterol diet did not change term non-laboring rat myometrial mitochondrial function [132]. We found no evidence for changes in myometrial cellular respiration, ROS with obesity, or effects of either on labor endurance.

## Discussion

**Fig. 2:**
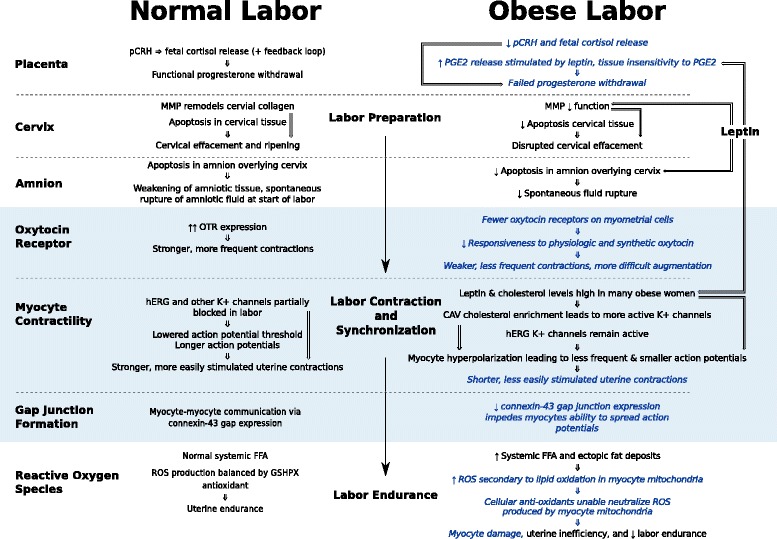
Comparison of normal and obesity-associated mechanisms of labor. MMP = matrix metalloproteinase, hERG K + =human ether-a-go-go-related gene potassium channel, pCRH = placental corticotropin releasing hormone, FFA = free fatty acid, ROS = reactive oxygen species, GSHPX = glutathione peroxidase, OTR = oxytocin receptor, PGE2 = prostaglandin E2, connexin-43 = myometrial gap junction. Blue italicized script = proposed mechanism or mechanism with limited evidence

In a theoretical model of successful human parturition, labor occurs as a result of “integrative and synergistic coordination” of separate biological processes or modules, occurring across many tissues [133]. With obesity, the delayed labor and labor dystocia phenotypes could result from multiple accumulated parturition malfunctions (Fig. 2) in the placenta, cervix, amnion, and myometrium. Obesity may also decrease labor endurance, with meta-inflammation and excess FFA causing excess myometrial ROS accumulation. Changes in biologic signals or responses with obesity could alter the onset, synchronization, and endurance of labor. Our literature search identified several questions that require additional basic and translational investigation, and as the effects of obesity are better understood, additional hypotheses will likely be generated. Considering the evidence for the pathobiology of labor dystocia in obesity, we suggest several investigative and therapeutic opportunities to improve vaginal delivery rates:

- *Basic science investigation:* We have summarized the existing evidence that obesity produces important changes in parturition signaling and the molecular program of labor in placenta, myometrium, and cervix. It is clear that each known step in the parturition process could be altered by hormones or other unique regulation produced by adipose tissue or associated meta-inflammation. However, we need to identify specific mechanisms.
  - o It will be important to define the type of estrogens (estriol or estradiol) produced in obese parturients and the effect of obesity on the ratio of estrogen to progesterone at term. In addition, the effect of obesity on placental hormone production and myometrial progesterone receptor expression must be quantified in both animal models and women if we are to develop new approaches to modifying the deleterious effects of obesity.
  - o Preclinical animal models of obesity management in pregnancy, for example limited weight gain and intentional weight loss, may reveal important parturition and fetal effects.
  - o Basic investigation of whether increased fat mass alone or associated metabolic syndrome and meta-inflammation drive parturition dysfunction is warranted. Metabolomic characterization of obese women with labor dystocia or Pitocin resistance could further our understanding of the signals linking obesity and labor outcome versus the separate influence of metabolic syndrome.
- *Clinical management:* Allowing additional time for obese women to complete cervical ripening and the first stage of labor before proceeding to cesarean delivery for slow labor progress could immediately improve outcomes [134, 135]. Clinical tools to identify aberrant labor progress with obesity, such as BMI-determined labor partograms, are needed. Further, investigating parturition changes with decreased weight gain or weight loss during obese pregnancy may be informative. The safety and outcome of altered prenatal and obstetric management requires thorough clinical evaluation and prospective trials [136].
- *Medication management:* Obese women may benefit from optimized protocols for Pitocin (and other induction/augmentation agents), rather than the universal protocols currently in use for women of any BMI. Higher doses and/or longer infusion times may be necessary for induction of labor in an obese woman. The differences in OTR expression and function and myometrial contractility between obese and normal-weight women are not yet defined. All induction agents may need to be examined to find the most effective regimen for obese cohorts.
- *Therapeutic investigation:*
  - o Cholesterol-lowering therapies (e.g., statins, niacin, bile acid sequestrants) might prevent hypercholesterolemia-linked suppression of contractions in obese women. Although statins are rated Category X in pregnancy, the teratogenic potential appears to be limited to extraordinarily high doses in animal models, and in select cases the potential benefits may outweigh the risks, even during pregnancy [137]. Randomized controlled trials (RCT) are needed.
  - o Thiazolidinedione (TZD) activates peroxisome proliferator-activated receptors (PPARs), allowing for safe storage of FFA, improved insulin sensitivity, and decreased cellular ROS damage [138]. Activation of the PPAR system is known to reduce recruitment of immune cells and inhibit inflammation, allowing better utilization of glucose as an energy source for cellular processes [65]. TZD therapy could improve labor endurance by decreasing myometrial ROS damage from FFA metabolism, although the known association of TZD with increased weight gain [139] could carry other risks. A RCT of TZD therapy to improve labor endurance is needed.
- *Nutrition intervention:*
  - o High-fat diet apparently decreases uterine blood flow [96] and lowers connexin-43 expression [129] in animal models. Prospective investigation of the effect of dietary fat in normal and obese women on labor length, induction response, and dystocia incidence is needed.
  - o Serum vitamin D is inversely related to both visceral and subcutaneous fat and insulin resistance [140], and significantly decreases biomarkers of oxidative stress [141]. A RCT investigating nutritional supplementation of anti-oxidants (e.g., vitamins D, C, E, and DHA/EPA) to modulate lipotoxicity-linked ROS elevation and improve labor outcomes is needed.

## Conclusion

Obesity is associated with changes in the placenta, cervix, amnion, and myometrium that could alter labor preparation, contraction/synchronization, and endurance. Studies investigating the biologic mechanisms of labor dystocia and failed induction due to obesity, both basic and translational, are needed. With a better understanding of the unique biology of obese parturients, we may develop better techniques and treatments to optimize labor outcomes, increase the rate of vaginal deliveries, and reduce the risk of obstetric complications. Even without new medications, changing our clinical management of obese women offers an immediate opportunity to decrease unplanned cesareans. Simply offering additional time in labor for obese women could result in increased vaginal deliveries. Therefore, while initial efforts to address obesity-related labor dysfunction could be directed to clinical management, future interventions can be designed to correct or overcome the altered parturition physiology in obese women. Moreover, as additional molecular details of human parturition are discovered, there may be substantial benefit to exploring the interactions with obesity. As obesity increases in the population, addressing the interactions between obesity and normal physiology is a women’s health priority, and may lead to important improvements in obstetric care and the well-being of mothers and their newborn children. For now, we suggest giving more time clinically to circumvent the parturition dysfunction in obesity, while we invest additional time and research in understanding better the pathobiological effects of obesity on normal parturition signaling.

## Additional file

Additional file 1: Table S1. Studies Located in Comprehensive Review of Mechanisms of Parturition Dysfunction in Obesity (PDF 956 kb)

## Abbreviations

ATP: adenosine triphosphate
ACTH: adrenocorticotropin hormone
BMI: body mass index
Ca^2+^: calcium ion
CAP: contractile associated protein
COX-2: cyclooxgenase-2 enzyme
CRH: corticotropin-releasing hormone
DHA/EPA: docosahexaenoic acid and eicosapentaenoic acid (omega-3 fatty acids)
DHEA: dehydroepiandrosterone
DM: diabetes mellitus
FFA: free fatty acid
GDM: gestational diabetes mellitus
GSHPx: glutathione peroxidase
hERG: human ether-a-go-go-related gene (potassium channel)
K^+^: potassium ion
LDL: low-density lipoprotein
Maxi-K channel: large conductance potassium channel
MMP: matrix metalloproteinase
OR: odds ratio
OTR: oxytocin receptor
pCRH: placental corticotropin-releasing hormone
PGE_2_: prostaglandin E2 (dinoprostone; naturally occurring prostaglandin)
PGF_2alpha_: prostaglandin F2alpha (dinoprost; naturally occurring prostaglandin)
PR: progesterone receptor
ROS: reactive oxygen species
PPAR: peroxisome proliferator-activated receptor
Pitocin: synthetic oxytocin
TZD: thiazolidinedione
