# Supplementary material for: Parturition dysfunction in obesity: time to target the pathobiology
Source: Reprod Biol Endocrinol. 2015 Dec 18;13:135. doi: 10.1186/s12958-015-0129-6 (PMC4683915; doi:10.1186/s12958-015-0129-6)
Supplement: Additional file 1: Table S1. — Studies Located in Comprehensive Review of Mechanisms of Parturition Dysfunction in Obesity (PDF 956 kb) [file 12958_2015_129_MOESM1_ESM.pdf]

Additional file: Table S1. Studies Located in Comprehensive Review of Mechanisms of Parturition Dysfunction in Obesity

Reference	Question of Study	Population	Intervention	Control	Observations	Significance
Labor Preparation Dysfunction in Obesity						
Farley et al, 2010 [97]	Do human placentas of OB women transport amino acids differently than those from non-OB women?	N=7 OB women (mean BMI 31.5) @ 39 weeks gestation	Measure baseline and leptin-stimulated sodium-dependent neutral amino acid transporter (SNAT) activity	N=4 lean women (mean BMI 22.4) @ 39 wks gestation	• Decreased SNAT activity in OB women's placentas (p=.005),<br>• Increased maternal hyperleptinemia among OB women (p=.001)	OB women significantly more hyperleptinemic and have placentas with decreased amino acid transport compared to placentas from normal controls.
Frias et al, 2011 [96]	Is abnormal uteroplacental circulation related to chronic high-fat diet in non-human primate model?	24 Japanese macaques fed a high fat diet for at least 4 years prior to study	Measure uteroplacental perfusion via ultrasound measurement in early 3rd trimester	24 macaques age-matched eating control diet, with similar reproductive histories	• Decreased uteroplacental perfusion (38% reduced) in high-fat animals that were insulin-sensitive. 56% reduced uterine flow in high-fat animals with insulin resistance.<br>• Increased insulin (4-5x higher) and leptin in high fat diet rats (5x higher).	High-fat diet decreases uterine blood flow. This effect is true when obesity is controlled for. Placental dysfunction is further deteriorated when both obesity and insulin resistance are present.
Jansson et al, 2013 [99]	Is placental amino acid transporter activity increased for pregnancies with large babies and obese women?	23 Swedish women with uncomplicated pregnancies and first trimester BMI 18.5-44.9 kg/m^2	Measure activity and protein expression of placental amino acid transporter systems A and L in syncytiotrophoblast microvillous plasma membranes.	Compare lower vs. higher maternal BMI	• Microvillous plasma membrane system A, but not system L, activity and protein expression of system A isoforms SNAT 2 positively correlated to birth weight ($p<0.001$).<br>• Insulin/IGF-I and mTOR signaling pathways increased in placentas of obese women having large babies.<br>• Birth weights (range 3,025-4,235g) positive correlation with maternal BMI ($P<0.05$)	Some amino acid transport isoforms are up-regulated among women with uncomplicated pregnancies and higher maternal BMI who had large babies.
Kramer et al, 2010 [100]	What upstream factors are associated with maternal CRH concentration at 24-26 weeks gestation?	205 socio-demographically diverse pregnant women with	Case control study nested in multicenter prospective cohort	430 control women	• Women with high (above median) plasma CRH less likely to be overweight or obese. OR 0.5 95% CI [0.3, 0.8]	Second-trimester maternal plasma CRH levels are significantly decreased in overweight and obese

Reference	Question of Study	Population	Intervention	Control	Observations	Significance
		spontaneous preterm birth			• Associations with maternal BMI persisted in regression analyses controlling for confounding factors	women compared to normal weight referent.
Konopka et al, 2013 [107]	When labor is induced with dinoprostone, what changes in maternal serum of progesterone, estradiol, and estriol can be measured?	Blood samples from 81 pregnant women at term, mixed weight.	Exploratory	None	• Progesterone levels decreased from admission to birth in pt who had successful induction of labor with dinoprostone.<br>• Women with failed dinoprostone induction did not have significant decrease of these hormones.	Successful cervical ripening with dinoprostone associated with reduced maternal progesterone from induction start till birth, reflecting functional progesterone withdrawal.
Lappas et al, 2005 [103]	What is the effect of leptin, resistin, and adiponectin on the release of proinflammatory mediators in placenta and adipose tissue from subcutaneous space?	Tissue samples from 5 women of placental and subcutaneous adipose tissue	Measure baseline and leptin, resistin, and Adiponectin exposed tissue release of IL-1 beta, TNFalpha, PGFalpha, and PGE2 with ELISA	Pre-adipokine exposed tissue	• No effect of resistin on proinflammatory cytokine or prostaglandin release<br>• Leptin at 100ng/ml and adiponectin at 0/1 mcg/ml sig increased release of IL-1beta, IL-6, TNFalpha, and PGE2 from human placenta and adipose tissue<br>• Leptin and Adiponectin-induced responses were abrogated following anti-inflammatory treatment	Leptin and adiponectin activate pro-inflammatory cytokine release from placenta and adipose tissue in humans, including PGE2 release.
Rosario et al, 2015 [98]	Does maternal obesity cause up-regulation of placental nutrient transporter expression in a mouse model?	Placental samples taken from N=80 multiparous C57BL/6J mice fed either control or high-fat/high-sugar pellet diet.	Measure maternal serum total adiponectin, leptin, insulin, cholesterol, non-esterified fatty acids, triglycerides, and glucose (after challenge).<br>Measure System A and L amino acid transporter activity in	Control group of mice fed normal pellet diet.	• High fat/High sugar mice had 25% increased fat mass, glucose intolerance, insulin, leptin, and cholesterol levels ($p<0.05$). Had lower Adiponectin levels. Had increased fetal weight (+18%, $p=0.0005$).<br>• Trophoblast plasma membranes from High	Maternal obesity in mouse model linked to increased expression of amino acid and glucose transporters in placenta, and these amino acid transport systems had greater activity along placental border.<br>This study unique in exposure of mouse model to diet high in fat AND high sugar, resulting in

Reference	Question of Study	Population	Intervention	Control	Observations	Significance
			trophoblast plasma membranes.		fat/High sugar mice placentas showed increased protein expression of glucose transporter 1 & 3, SNAT 2, and large neutral amino acid transporter 1.	obesity. This contrasts with other animal studies not showing animal obesity after high fat high cholesterol diets.
Stirrat et al, 2014 [101]	How are CRH and other hypothalamic-pituitary-adrenal hormones associated with pregnancy outcomes in obese women?	286 obese (BMI 44.05 +/- 3.98kg/m^2) pregnant women	Measure CRH by radioimmunoassay in venous blood at 16, 28, and 36 weeks of gestation, labor onset gestational age, birthweight of baby	137 lean (BMI 22.71 +/- 1.66 kg/m^2) pregnant women	• CRH was significantly lower in obese women at all time points ($p<0.05$)	Decreased HPA axis activity, including CRH activity, in obese pregnancy may be a mechanism for prolonged pregnancy.
Suidan et al, 2014. [104]	Compare labor outcomes in obese women having labor induction with misoprostol vs. dinoprostone	564 obese women, 267 exposed to dinoprostone	Retrospective review of patients at single institution 2008-2013	297 obese women having induction in same institutions exposed to misoprostol	• Misoprostol group had more successful cervical ripening (OR 1.79 [1.23-2.6]) compared to dinoprostone<br>• Significance of misoprostol success persisted after adjustment for parity, gestational age, birth weight, and indication for IOL	Obese women having induction of labor are more responsive to misoprostol (PGE1) for cervical ripening when compared to dinoprostone (PGE2). Reflect possible baseline elevation of PGE2 in obese women leading to less sensitivity.
Wallace et al, 2012 [95]	Is placenta weight and efficiency associated with maternal BMI?	55,105 placentas from births in Scotland, 1976-2007	None<br>Retrospective cohort study	Normal weight referent (BMI 18.6-24.9)	• Placental weight increased with BMI<br>• Placental hypertrophy increased with BMI<br>• Ratio of fetal/placental weight (placental efficiency) lower ($p<.001$) in OW, OB, and morbid OB women when compared to normal and underweight women	Placentas from OB women have reduced placental efficiency compared to those from normal-weight women.
Wendremaire et al, 2011 [109]	What is the role of leptin in human myometrium with inflammation-induced apoptosis?	Human myometrial specimens from 22 women having elective CS at 38-40 weeks prior to labor. Women	Measured amount of LPS-induced apoptosis in myometrial strips after a 60-minute incubation of the strips with leptin (in	Control myometrial biopsies taken from same or similar women, not exposed to leptin.	• Leptin receptors expressed in myometrial tissue seen by immuno-staining.<br>• Leptin prevents LPS-induced apoptosis in human myometrial tissue	Leptin in vitro exposure diminishes apoptosis in human myometrial tissue. This effect could change preparation of myometrium for labor and decrease necessary weakening of

Reference	Question of Study	Population	Intervention	Control	Observations	Significance
		categorized by pre-pregnancy BMI	physiologic ranges of 10^{-10}, 10^{-9}, and 10^{-8} mol/L) 48 hours before LPS treatment. Used both DAPI solution staining experiments and Western blot to quantify amount of apoptosis.		in a dose-dependent manner, decreasing pro-apoptotic markers (cleaved caspase-3, $p < .05$ and BAX, $p < .001$) and increasing antiapoptotic BCL2 ($p<0.05$)	fetal membranes for spontaneous rupture in labor.
Wendremaire et al, 2012 [108]	Does leptin interfere with MMP activity in human myometrium?	Human myometrial biopsies from women between 38-40 weeks by CS, prior to labor.	Measured total collagen content and MMP activity after incubation of myometrial biopsies in different concentrations of leptin	Control myometrial biopsies taken from same or similar women, not exposed to leptin.	• Total collagen content in stained tissue increased ($p<.001$) after leptin exposure.<br>• MMP activity and expression decreased ($p<.001$) in biopsies exposed to leptin when compared to controls.<br>• Effects abolished in myometrial samples exposed to pre-treatment with leptin receptor antagonist.	Leptin in vitro exposure blocks MMP degradation of collagen in myometrial tissue.
Wendremaire et al, 2013 [151]	Does leptin interfere with lipopolysaccharide-induced collagen degradation in human myometrium?	Human myometrial biopsies from women between 38-40 weeks by CS, prior to labor.	Measured total collagen content and MMP activity after incubation of myometrial biopsies in different concentrations of leptin	Control myometrial biopsies taken from same or similar women, not exposed to leptin.	• Leptin prevented decrease in myometrial collagen content in a dose-dependent manner.<br>• Collagen synthesis also stimulated by leptin.	Leptin in vitro exposure stimulates collagen synthesis, which is normally down regulated in preparation for labor

Labor Contraction/Synchronization Dysfunction in Obesity						
Cluff et al, 2006 [58]	How do connexin 43 and syndecan 3 mRNA expression and protein distribution in human uterine tissue differ during normal vs. prolonged labor?	Uterine biopsies from nonpregnant (n=7), term pregnant women not in labor (n=14), in normal labor (n=7), and prolonged labor (n=7). Mixed weight samples.	Measured mRNA levels of syndecan 3 and connexin 43 using real time RT-PCR	Biopsies from non-pregnant, term pregnant women not in labor, and women in normal labor	• mRNA expressions of Connexin-43 and syndecan 3 lower in women with prolonged labor vs. those in normal labor (p< 0.005)<br>• Connexin-43 distribution in uterine tissue was weaker and more unevenly distributed in women with prolonged labor vs. those with normally-progressing labor	Women of mixed weight with prolonged labor patterns show decreased mRNA expression and more uneven/less strong distribution of connexin-43 and syndecan 3 compared to women with normally-progressing labor. Obese women with prolonged labor may show similar distributions.
Elmes et al, 2011 [129]	Does a HFHC diet decrease markers of uterine contractility during parturition in the rat?	N=10 Wistar rats fed a HFHC diet for 6 weeks followed by mating and maintenance of diet during pregnancy	Measured body and fat deposits at onset of labor. Myometrial tissue analyzed for cholesterol, triglycerides, and expression of connexin-43.	N=10 control rats fed a normal chow diet for 6 weeks prior to mating and during subsequent pregnancy.	• HFHC rats gained greater weight than controls (p<.003) and had greater increases in peri-renal fat (p<.01).<br>• Total cholesterol, triglyceride levels similar in two groups.<br>• Connexin-43 expressed significantly less in myometrial tissue from HFHC rats (p=.059).	Connexin-43 expression reduced in HFHC diet rats. Unable to observe any effects of rat labor or myometrial contractility in this experimental design.
Fyfe et al, 2013 [123]	Does early pregnancy serum cholesterol predict first stage cesarean for failure to progress at term?	N=1,036 term, nulliparous, BMI >25kg/m2	Serum cholesterol levels at 14-16 weeks gestation	Subjects with first stage failure to progress (n=196) compared to vaginal birth (n=840)	• Total cholesterol at 14-16 gestational weeks was not significantly different among term, nulliparous women having cesarean for failure to progress vs. those having vaginal delivery (5.55 +/- 0.92 versus 5.67 +/- 0.85 mmol/L, p=0.10)	Early pregnancy serum cholesterol not associated with CD for indication of failure to progress in nulliparous, term women.
Garabedian et al, 2011 [126]	Does BMI correlate with expression of OTR and Connexin-43?	20 human myometrial specimens obtained from women undergoing planned, pre-	Measured mRNA expression of OTR and connexin-43. Measured BMI at delivery of women myometrial donors.	Normal maternal BMI referent samples	• OTR expression correlated with BMI at delivery (p=0.004) among women having primary CS.<br>• Connexin-43 expression not correlated with	OTR gene expression reduced in term, non-laboring human myometrial tissue with higher maternal BMI. No difference in Connexin-43 gene by maternal BMI.

		labor repeat CS (n=13) or primary CS (n=7) at term.			maternal BMI at delivery.	
Grotegut et al, 2013 [127]	Is myometrial OTR gene and protein expression affected by obesity in pregnancy?	63 human myometrial samples from term and preterm (mean gestational age=38.0 [33.0, 39.1 quartiles] women at CS. 33.3% labored prior to CS.	OTR mRNA PCR and OTR protein Western Blot.	Normal maternal BMI referent samples	• OTR gene expression not correlated with maternal BMI at delivery (r^2=.023, *p*=.250).<br>• Mean relative OTR protein expression also did not differ between BMI < 30 kg/m^2 compared to BMI > 40kg/m^2 (p=.938 for difference in means).	No change in OTR gene or protein expression by maternal BMI in term human myometrial tissue from mixed sample of laboring (1/3 of sample) and non-laboring women.
Hehir, Glavey & Morrison, 2008 [78]	What are the effects of ghrelin on human myometrial contractility in vitro?	21 human myometrial strips from women with elective CS between 38-39 weeks.	Physiologic solution organ bath exposed to cumulative ghrelin concentrations (10^{-9} mol/L-10^{-6} mol/L). Both spontaneous and OXY-induced CTX measured.	Non-exposed myometrial strips taken from the same biopsies	• Ghrelin had inhibitory effect on contractility (33.66% +/- 2.63% for spontaneous CTX, 31.55% +/- 4.64% for OXY-CTX.	Ghrelin, found at lowest levels among obese women, and at lowest levels near term, may inhibit contractility at term. Unknown interaction in labor with obese women.
Hehir and Morrison, 2012 [73]	What is the effect of apelin on human uterine contractility in vitro?	17 human myometrial strips from women with elective CS between 38-39 weeks.	Physiologic solution organ baths, exposed to cumulative apelin concentrations (1nmol/L to 1 µmol/L). Both spontaneous and OXY-induced CTXs measured.	Non-exposed myometrial strips taken from same human biopsies	• Apelin inhibited spontaneous (36.8 ± 6.4%, n=6, p= .002) and OXY-induced CTXs (30.4 ± 4.6%, n=6, p < .0001).<br>• Frequency and amplitude of CTXs reduced.	In vitro apelin reduces frequency and amplitude of spontaneous and OXY-induced human myometrial CTXs.
Higgins et al, 2010 [152]	What is the relationship between BMI and the ability of the myometrium to contract spontaneously and in response to OXY in an in vitro model?	609 human myometrial strips from 85 nonlaboring women with term gestation of known early pregnancy BMI.	Physiologic organ baths, exposed to synthetic OXY. Contractility data (frequency, amplitude, integral activity) recorded via tension transducer.	Myometrial strips taken from lean women compared to those taken from OB women.	• No correlation between maternal early pregnancy BMI and spontaneous myometrial contractile activity.<br>• Myometrial response to the addition of synthetic OXY in tissue bath was not affected by donor's BMI.	Spontaneous CTX in human myometrial tissue from OB and OW not different. This finding contrasts with other human myometrial tissue investigations showing reduction in CTXs with in vitro exposure to cholesterol, apelin, leptin, and LDL.

Mouzat et al, 2007 [122]	What is the phenotype in mice of cholesterol esters accumulation in the uterus?	Uteri from 3-month old mice during metaestrus.	Measurement of lipids via chromatography in uteri. Protein and RNA, as well as measurement of in vitro uterine contractions performed.	Uteri from mice exposed to same protocols, yet with normal cholesterol ester efflux ability (LXRbeta inact)	• LXRbeta regulates cholesterol efflux within myocyte cells<br>• When cholesterol esters accumulate in myocytes, contractions become lower amplitude<br>• Mice engineered to accumulate cholesterol in their myometrial cells have difficulty in pregnancy, showing nonexpulsed pups in uterine horns.	Mice with dysfunctional liver clearance of liver cholesterol exhibit reduced oxytocin responses and abnormal labors
Moynihan et al, 2006 [112]	What are the effects of leptin on human uterine contractility in vitro?	18 human myometrial strips from women at elective (prelabor) CS.	Physiologic organ baths, exposed to cumulative concentrations of leptin (1nmol/L to 1 μmol/L). Both spontaneous and OXY-induced CTXs measured.	Controls were myometrial strips taken from same human biopsies, placed in same physiologic baths.	• Leptin inhibited spontaneous (inhibition of 46.794 ± 5.1333%, n=6, $p < .001$) and OXY-induced CTXs (inhibition of 42.323 ± 3.692%, n=6, $p < .001$).<br>• Frequency and amplitude of CTXs were reduced.	Leptin in-vitro exposure causes decreased spontaneous and OXY-induced contraction frequency, duration, and amplitude in human myometrial tissue.
Mumtaz et al, 2015 [76]	What are the effects of visfatin and leptin on myometrial contractility in both rat and human?	Biopsies of human myometrial tissue from 22 non-laboring women between 38-40 gestational weeks, BMI 23-35.<br>Rat myometrial tissue of late pregnancy.	Physiologic organ baths, exposed to visfatin or leptin and analyzed for amplitude and force of contraction. Both spontaneous and OXY-induced CTXs measured.	Controls were myometrial strips taken from the same rats or human biopsies, placed in same physiologic baths.	• Visfatin showed dose-dependent decrease in contractility in both human and rat tissue.<br>• Compared to leptin, visfatin showed more potent effects of CTX in both rat and human.	Visfatin inhibits myometrial contractility more potently than leptin. Increased visfatin in obese pregnant women may impair uterine contractility during labor.
Parkington et al, 2014 [60]	Does elevated BMI change myometrial potassium channel (hERG) function and CTX amplitude/duration before and during labor?	Human myometrial strips from OB women at the time of CS (BMI > 30 kg/m^2). Some in labor and some not.	Physiologic organ baths to measure CTX and action potential characteristics. Dofetilide solution used to create hERG blockade. Whole-cell patch clamp to	Myometrial strips from lean women (BMI < 30kg/m^2), some in labor and some not	• hERG potassium channels shorten duration of myometrial action potential plateau.<br>• hERG channels partially blocked in myometrial tissue from laboring lean women by hERG ß-subunit.	Failure of hERG ß-subunit blockade of hERG potassium channel function in laboring OB women causes hyperpolarization of myocytes, shorter action potentials, and decreased action potential plateau phase.

			evaluate hERG channel activity. Western blot analysis to quantify amounts of hERG subunit in tissues.		• hERG ß-subunit expression decreased in laboring OB women, causing continued hERG suppression of CTX.<br>• Action potentials in laboring OB women shorter and more spiked than those in non-OB women.	hERG findings may be mechanistically linked to investigations showing decreases of CTX by high cholesterol environments--hERG and other potassium channels located on plasma membrane lipid rafts, enhanced by increased cholesterol.
Smith et al, 2005 [120]	Does cholesterol alter uterine contractility?	Rat myometrial strips from late pregnancy (19-21 days)	Physiologic solution organ bath with bilateral clips to measure force and epifluorescent microscope to measure changes in intracellular Ca^{2+}. Cholesterol added to myometrial perfusate, then MCD (methyl-ß-cyclodextrin) solution added to measure effects of cholesterol removal.	Control strips taken from same rats, exposed to same solutions except cholesterol addition.	• Increasing cholesterol to baths decreased CTX frequency (0.72 ± 0.05/min control to 0.4 ± 0.04/min or 0.22 ± 0.05/min in two cholesterol baths), duration (28.3 ± 2.0 sec control vs. 16 ± 1.2 sec with cholesterol) and amplitude (92% ± 34.1% and 68 ± 2.45% of control in cholesterol baths).<br>• MCD addition to the baths removed cholesterol and reversed these changes. LDL addition to baths had same effect as cholesterol.	Cholesterol and LDL in-vitro exposure causes decreased contraction frequency, duration, and amplitude in rat myometrial tissue. Removal of cholesterol from myometrial tissue reverses these effects.
Zera et al, 2010 [113]	Do second trimester leptin levels predict cesarean delivery for failed first stage labor independent of maternal BMI?	Retrospective chart review and secondary analysis of 1,512 women	none	Women in same cohort study	• Among the 87 women who had cesarean for failed first stage labor, mean leptin levels were higher than those without this outcome (26.5 vs. 23.2, p=0.03)<br>• Leptin was significantly associated with cesarean for failed first stage labor among nulliparous women in model with maternal age, race,	Second trimester serum leptin levels did not explain nulliparous or multiparous women's risk for failed first stage labor in an adjusted multivariate model that included maternal BMI.

					education, smoking, household income, birthweight (OR 1.14 per 10ng/mL, [0.96-1.37].<br>• After adjustment of model for maternal BMI, OR was not significant, OR=0.99 [0.80-1.24]	
Zhang et al, 2007 [121]	Does cholesterol inhibit myometrial contractility and Ca^{2+} signaling? Is addition of synthetic OXY adequate to augment CTXs in a high-cholesterol environment?	97 Human myometrial strips:<br>n=36 from non-pregnant women<br>n=30 from term pregnant nonlaboring women,<br>n=28 from term pregnant laboring women.<br>BMI of women not reported.	Physiologic organ bath to measure force and simultaneous Ca^{2+} of myometrial CTXs. Cholesterol added to myometrial perfusate (concentration of 2.5, 5 or 10 mg/mL), followed by MCD. LDL used in other baths.	Myometrial strips from same donors, exposed to same organ bath solutions with exception of cholesterol and LDL.	• Dose-dependent reduction in CTX amplitude with cholesterol exposure (93% ± 4%, 84% ± 2%, and 35% ± 5% of control amplitude)<br>• Reduction in CTX duration (3.3 ± 0.5 minutes control vs. 2.1 ± 0.3 minutes in cholesterol 10mg/mL solution).<br>• LDL addition to baths with similar effects.<br>• Addition of MCD to solutions restored and reversed these reductions.<br>• OXY-induced CTXs were also reduced (amplitude, duration) by cholesterol and LDL addition to baths.<br>• Changes seen in response to cholesterol were associated with changes in Ca^{2+} in myometrial strips.	Cholesterol and LDL in-vitro exposure causes decreasing contraction duration and amplitude in human myometrial tissue. These CTX changes mirror decreases in Ca^{2+} flow across myometrial tissue. Effects not reversed by OXY addition. Removal of cholesterol from myometrial tissue reverses these effects.
Labor Endurance Dysfunction in Obesity						
Gam et al, 2014 [132]	Does maternal obesity change oxidative capacity and/or morphology of rat myometrial mitochondria?	20 Hannover Wistar rats fed 47 days prior to gestation and during gestation with a HFHC diet	Function and morphology of myometrial mitochondria examined at full term	10 rats of same breed fed regular chow diet	• Fasting plasma FFA, cholesterol levels, and daily energy intake not different by diet type.<br>• HFHC rats had higher fat percentage compared to	High fat and high cholesterol diets do not cause changes in term, non-laboring rat myometrial mitochondria functioning.

		or a high fat, low carbohydrate diet			regular chow rats (p< .05).<br>• Maximal oxygen consumption, mitochondria ATP-production efficiency, and amount of mitochondria per gram of myometrium did not differ between groups.	
Mele et al, 2014 [131]	Does maternal obesity change mitochondrial function in human placentas?	Human placental tissue from women with pre-pregnancy BMI ranging from 18.5-45 following pre-labor CS.<br>n=6 samples from women BMI 25-29.9<br>n=6 samples from OB women (BMI>30)	Measured placental ATP generation, mitochondrial biogenesis, expression of electron transport chain subunits, and mitochondrial function	Six placental samples from lean women (BMI 18.5-24.9)	• Increased pre-pregnancy BMI associated with term placentas showing excessive ROS and reduction in ATP levels.<br>• Mitochondrial respiration in trophoblast taken from OB women was reduced, and showed reduced flexibility to respond beyond basal metabolism.	Pre-pregnancy OB associated with excessive ROS production and decreased ATP generation by term human placental mitochondria.
Quenby et al, 2004 [130]	Does in vivo myometrial acidification contribute to dysfunctional labor?	Blood samples taken from lower uterine segment of uterus from women have CS for either dysfunctional labor or elective.<br>Myometrial strips also collected.	Analyzed blood samples for pH, O2 saturation, lactate levels.<br>Myometrial strips from elective CS exposed to pH from normal and dysfunctionally-contracting uteri.	Blood samples from women having elective CS.<br>Myometrial strips exposed to pH of normal labor.	• pH from uterus of women with dysfunctional labor was significantly lower than that from women having elective CS (7.35 vs. 7.49) or CS with normal CTX (7.47).<br>• Myometrial strips exposed to pH 7.3 solution showed more irregular CTX compared to strips exposed to pH 7.5.	Lower pH in the myometrium during labor may lead to labor dystocia.

OB=obese, OW=overweight, LPS=lipopolysaccharide, CS=cesarean section, OTR=oxytocin receptor, PCR=polymerase chain reaction, mRNA=messenger RNA, BMI=body mass index, LDL=low density lipoproteins, hERG=Human ether-a-go-go related gene, DAPI=diamidino-2-phenylindole solution, OXY=oxytocin, HFHC=high fat high cholesterol diet, CTX=contraction, ATP=adenosine triphosphate, O2=oxygen, FFA=free fatty acids, MCD=methyl-B-cyclodextrin, chemical for depleting cholesterol from cell membranes, Ca2+=calcium, CRH=corticotrophin releasing hormone, TNFalpha, IL-1beta, IL-5=cytokines, SNAT=sodium-coupled neutral amino acid transporter
